# Addressing Malnutrition in Children: An Appraisal of the Ondo State Nutrition Program

**DOI:** 10.7759/cureus.28595

**Published:** 2022-08-30

**Authors:** Olugbenga O Osunmakinwa, Victoria O Oladoyin, Oluyemi Okunlola, Demilade Ibirongbe, Oyetunde T Oyeyemi, Ajoke Awosusi, Olalekan W Adebimpe, Olorunfemi Ogundele, Jumoke Akinkuotu, Kikelomo Adejuwon

**Affiliations:** 1 Community Medicine, University of Medical Sciences, Ondo, NGA; 2 Basic Medical Sciences, University of Medical Sciences, Ondo, NGA; 3 Biological Sciences, University of Medical Sciences, Ondo, NGA; 4 Human Kinetics and Health Education, Ekiti State University, Ado-Ekiti, Ado Ekiti, NGA; 5 Directorate of Nutrition and Health Promotion, Ondo State Primary Health Care Development Agency, Ondo, NGA

**Keywords:** nigeria, ondo state, nutritional status, under five children, ndhs, comprehensive nutrition program

## Abstract

Background

The report of the 2013 Nigeria Demographic and Health Survey (NDHS 2013) showed Ondo State had one of the worst indices for stunting and underweight in the southwestern geopolitical zone of the country, a development that was considered "unacceptable" by the state government. In the bid to reverse the ugly trend, the State Primary Health Care Development Agency put in place a comprehensive nutrition program in 2015 tagged "Nutrition Rebirth," aimed at reversing the high prevalence of malnutrition among under-five children in the State.

Aims

This study seeks to evaluate the Ondo State comprehensive nutrition program by comparing the children's nutritional status pre- and post-period under review in the implementation of the program. This provides a measure of assessment of the performance of the program as implemented in the period under review.

Materials and methods

This study covered the period between 2015 and 2017 in the implementation of the nutrition rebirth program. Data for the study were obtained from the NDHS (2013) and NDHS (2018) nutrition of children and women data. Analysis of the NDHS (2013) and NDHS (2018) data commenced simultaneously with field work and lasted for about six and eight months, respectively. Analysis of the data obtained from the NDHS for this study spanned through a period of about three weeks. An ecologic time-trend analysis was used to compare the trends in nutritional status indicators among under-five children in all 18 Local Government Areas (LGAs) in Ondo State.

Results

Malnutrition among children less than five years dropped in most of the LGAs in 2018. Malnutrition was not associated with children's gender and maternal age in 2013 and 2018. Prevalence of stunting growth and underweight was associated with maternal education, wealth index, residence type, place of delivery, and LGA in 2013 (p < 0.05) but was not in 2018 (p > 0.05).

Conclusions

The spatial analysis of the Ondo State comprehensive nutrition program showed that the program has a positive impact, reducing malnutrition among children under five years; but critical appraisal of implementation challenges in LGAs with no significant reduction in malnutrition among their children under five years is recommended. The comprehensive and wholistic approach of the program is also recommended for other states and settings with a high prevalence of under-five malnutrition to understudy the possible adaptation, as appropriate.

## Introduction

Background

Malnutrition, defined as deficiencies or imbalances in the intake of nutrients, is among the leading cause of death in children below age five in developing countries [1]. Two forms of this disorder, with fatal and other serious devastating effects, exist, namely, overnutrition and undernutrition [1-5]. Undernutrition, categorized into stunting, wasting, and underweight, can be identified with anthropometric measurements. Stunting, otherwise known as low height-for-age or chronic undernutrition, is a growth retardation problem occurring as a result of long-term nutritional deprivation [6]. Wasting, otherwise known as low weight-for-height or acute undernutrition, on other hand, results from acute food shortage or the presence of a disease. The third indicator for measuring undernutrition in children is underweight, which is also referred to as low weight-for-age. This reflects both acute and chronic undernutrition [6].

The Nigeria Demographic Health Survey (NDHS) 2013 report showed that Ondo State had one of the worst indices for stunting and underweight in the southwestern geopolitical zone of the country [7]. As a result of these frightening statistics in Ondo State, a comprehensive nutrition program tagged "Nutrition Rebirth" was implemented between 2015 and 2017 (the period under study) by the Ondo State government to curb the menace of malnutrition in the State. The nutrition program took a wholistic approach to tackle the unacceptably high pre-intervention prevalence of undernutrition among under-five children in the State, which was estimated to be 24% for stunting, 6.6% for wasting, and 13.4% for underweight according to the 2013 NDHS data [7].

Activities

Activities carried out in the program included procurement of relevant nutrition, growth-monitoring equipment, and materials for all primary health facilities in Ondo State; routine growth monitoring; vitamin supplementation and distribution of zinc sulfate; live educational program on exclusive breastfeeding and consumption of appropriate diet on the State’s radio stations (Positive FM 102.5 and Ondo State Radiovision Corporation/OSRC 96.5 FM); zonal training of health workers on infant and young child feeding as well as on International Code of Marketing of Breast Milk Substitutes; conduct of regular supportive supervision on key nutritional interventions in the State; partnership with some formula food companies on reduction of protein energy malnutrition in the local government areas (LGAs), and monthly feedback meeting of the Ondo State Primary Health Care Development Agency (OSPHCDA) with the 18 LGA nutrition focal officers. Another important component of the Ondo State nutrition program was the production of blended complementary foods (BCF) sourced from locally available and affordable food materials for the management of moderate acute malnutrition (MAM) cases.

This appraisal will help in determining how well the program is doing and possibly recommending the same to other states in the country with malnutrition problems among children under five years. This study, therefore, aimed to evaluate the effect of the Ondo State comprehensive nutrition program (nutrition rebirth) on the nutritional status indicators of children under five years of age in Ondo State, using the NDHS data.

## Materials and methods

We used an ecologic, time-trend analysis to compare the trend in nutritional status indicators among under-five children in all 18 LGAs in the Ondo State. Data for the study were obtained from the NDHS (2013) and NDHS (2018) nutritional status of children and the sociodemographic data of women. Data for a total of 523 and 177 under-five children in the NDHS (2013) and NDHS (2018), respectively, with valid and complete information on date of birth, height (in centimeters), and weight (in kilograms) were analyzed for Ondo State and were utilized for this study [7].

The 2013 and 2018 NDHS data on nutrition were collected using the NDHS household questionnaire. Measurements of height and weight were obtained for children born in the five years preceding the surveys in all of the selected households. Weight measurements (in kilograms) were made using lightweight SECA GmbH scales (with digital screens), while height and length (in centimeters) were measured using a ShorrBoard® measuring board. Children under 24 months were measured lying down on the board, and standing height was measured for all other children [7,8].

To assess the precision of measurements, one child per cluster was randomly selected to be measured a second time. A difference of one centimeter or less between the two height measurements was defined as an acceptable level of precision. Children with a Z-score of less than -3 or more than 3 for height-for-age, weight-for-height, or weight-for-age were flagged and measured a second time. Re-measurement of flagged cases was performed to ensure accurate reporting of height and weight measurements [8]. The interviewers for the NDHS were trained on the use of the data collection tool and anthropometric measurements.

For this study, the 2013 and 2018 NDHS dataset was downloaded from the MEASURE DHS database after permission was granted. Thereafter, Ondo State data for all children under five years were extracted from the NDHS 2013 and 2018 and saved in the Statistical Package for Social Sciences (SPSS) software version 23 data file (IBM Corp., Armonk, NY).

Cross-tabulations were done using the Chi-square test to determine if the populations used for the two datasets were similar. The mean and standard deviations of the ages of the children and their mothers for the two data sets were also compared using the student's t-test. The height-for-age Z-score, weight-for-height Z-score, and weight-for-age Z-score for each dataset were calculated using the WHO Anthro software version 3.2.2. The Z-score for each nutritional status indicator was dichotomized into below minus two standard deviations (<-2 SD) and minus two standard deviations and above (≥-2 SD). Children with height-for-age Z-score < -2 SD were considered stunted, children with weight-for-height Z-score < -2 SD were considered wasted, while children with weight-for-age Z-score < -2 SD were considered underweight. The percentage of children with Z-scores < -2 SD for each of the nutritional status indicators were compared between the Ondo State 2013 and 2018 NDHS data across the 18 LGAs in the State. Permission to use the 2013 and 2018 NDHS dataset was obtained from MEASURE DHS.

## Results

The sociodemographic characteristics of the sampled children and women were presented in Table 1. A total of 560 and 219 children were sampled in 2013 and 2018, respectively. There was no statistically significant difference (PV = 0.941) between the mean age ± SD (in months) of children sampled in 2013 (28.66 ± 17.614) and 2018 (28.77 ± 17.567). Similarly, the size of children at birth, maternal age, and residence showed no statistically significant difference between the proportion of children sampled in 2013 and 2018, with p-values of 0.116, 0.812, and 0.099, respectively. However, there was a statistically significant difference in maternal primary education (PV = 0.000) and middle-class wealth ranking (PV = 0.000) among the mothers of sampled children in 2013 and 2018. Also, a statistically significant difference was observed in the place of delivery of sampled children with a greater proportion of mothers delivering in government health centers in 2018 (112 [64%]) compared to 2013 (63 [36%]), PV = 0.000 (Table 1).

**Table 1:** NDHS year by sociodemographic characteristics of sampled children and their maternal information NDHS: Nigeria Demographic and Health Survey; SD: Standard deviation; LGA: Local government area; ---: Nil data reported.

	NDHS Year		P-value
	2013 Frequency (%)	2018 Frequency (%)	
	N = 560	N = 219	
Gender			0.114
Male	269 (69.3)	119 (30.7)	
Female	291 (74.4)	100 (25.6)	
Age (in months)			0.714
0-5	58 (69.9)	25 (30.1)	
6-11	77 (77.8)	22 (22.2)	
12-23	96 (69.6)	42 (30.4)	
24-35	112 (73.2)	41 (26.8)	
36-47	106 (69.3)	47 (30.7)	
48-60	111 (72.5)	42 (27.5)	
Mean age ± SD (in months)	28.66 ± 17.614	28.77 ± 17.567	0.941
Size at birth			0.116
Very large	44 (68.8)	20 (31.3)	
Larger than average	208 (68.9)	94 (31.1)	
Average	260 (75.1)	86 (24.9)	
Smaller than average	37 (78.7)	10 (21.3)	
Very small	11 (55.0)	9 (45.0)	
Maternal age (in years)			0.812
15-19	13 (61.9)	8 (38.1)	
20-24	79 (73.8)	28 (26.2)	
25-29	149 (70.6)	62 (29.4)	
30-34	134 (70.5)	56 (29.5)	
35-39	116 (73)	43 (27)	
40-44	47 (73.4)	17 (26.6)	
45-49	22 (81.5)	5 (18.5)	
Maternal mean age ± SD (in years)	31.14 ± 6.713	30.58±6.366	0.281
Maternal highest educational level			0.000
No education	77 (82.8)	16 (17.2)	
Primary	172 (79.3)	45 (20.7)	
Secondary	247 (64.7)	135 (35.3)	
Higher	64 (73.6)	23 (26.4)	
Wealth index			0.000
Poorest	0 (0.0)	12 (100.0)	
Poorer	132 (79.5)	34 (20.5)	
Middle	135 (66.5)	68 (33.5)	
Richer	147 (70.3)	62 (29.7)	
Richest	146 (77.2)	43 (22.8)	
Residence			0.099
Urban	227 (68.8)	103 (31.2)	
Rural	333 (74.2)	116 (25.8)	
Place of delivery			0.000
Respondent's home	109 (79.6)	28 (20.4)	
Other home	167 (100)	0 ()	
Government hospital	136 (80.5)	33 (19.5)	
Government health center	63 (36)	112 (64)	
Government health post	4 (100)	0 ()	
Private hospital/clinic	81 (75.7)	26 (24.3)	
Other	0 ()	20 (100)	
LGA			0.000
Akoko North East	27 (47.4)	30 (52.6)	
Akoko North West	21 (52.5)	19 (47.5)	
Akoko South East	0 ()	13 (100)	
Akoko South West	13 (36.1)	23 (63.9)	
Akure North	43 (86)	7 (14)	
Akure South	46 (86.8)	7 (13.2)	
Ese Odo	0 ()	7 (100)	
Idanre	113 (100)	0 ()	
Ifedore	27 (67.5)	13 (32.5)	
Ilaje	57 (87.7)	8 (12.3)	
Ile Oluji/Okeigbo	53 (82.8)	11 (17.2)	
Irele	0 ()	6 (100)	
Odigbo	47 (75.8)	15 (24.2)	
Okitipupa	15 (57.7)	11 (42.3)	
Ondo East	---	---	
Ondo West	14 (56)	11 (44)	
Ose	61 (79.2)	16 (20.8)	
Owo	23 (51.1)	22 (48.9)	

**Figure 1:**
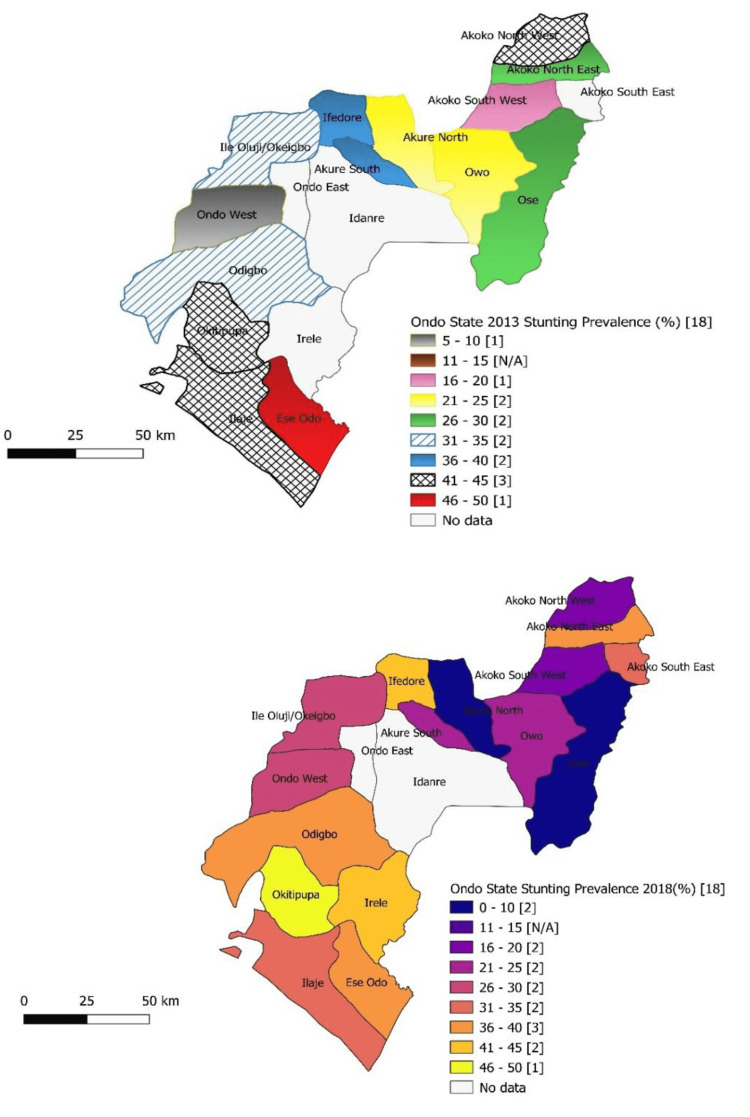
Map of the Ondo State showing a prevalence of stunting among under-five children by local government in 2013 and 2018 N/A: No Local government area in this category.

Stunting among children in Ese Odo, Ose, and Akure North LGAs with a prevalence range of 46%-50%, 26%- 30%, and 21%-25% in 2013 dropped to 36%-40%, 0%-12%, and 0%-10%, respectively, in 2018. No change was recorded in the stunting status among children in Owo (21%-25%) and Akoko South-West (16%-20%) LGAs between 2013 and 2018. The prevalence of stunted growth in children in Ondo West LGA ranged between 5% and 10% in 2013 but increased to 26% and 30% in 2018. An increase in the prevalence of stunted growth among children was also recorded in Odigbo (31%-35% to 36%-40%), Ifedore (36%-40% to 41%-45%), and Okitipupa (41%-45% to 46%-50%) LGAs (Figure 1).

**Figure 2:**
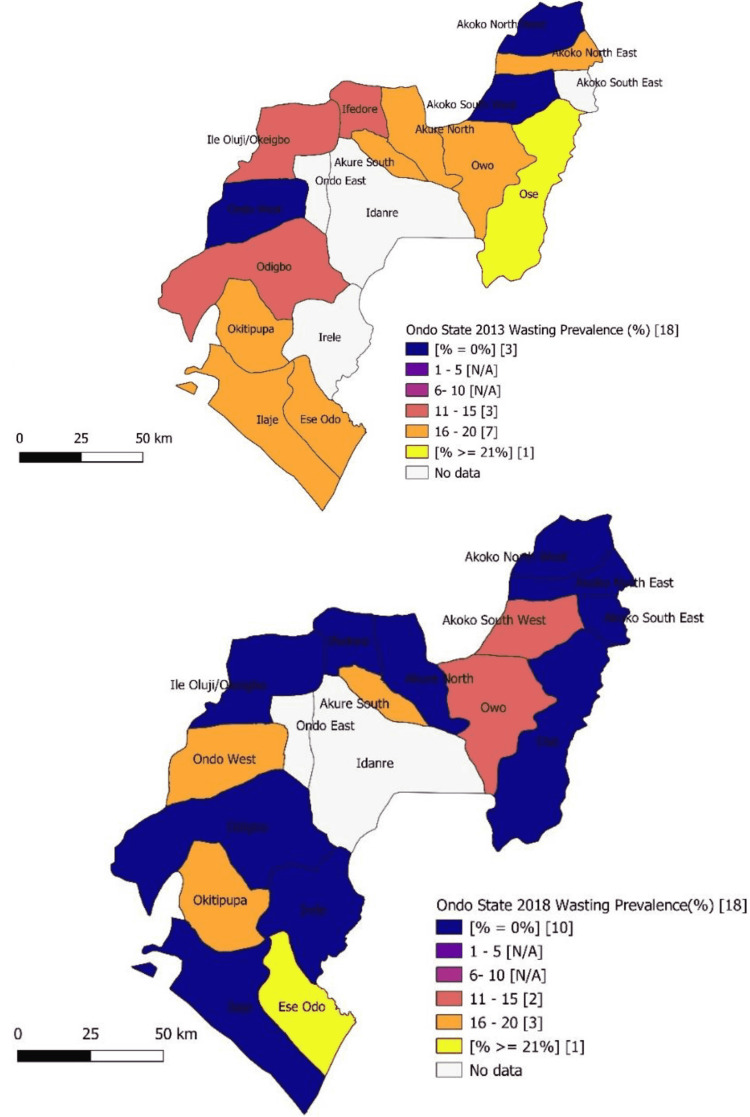
Map of the Ondo State showing a prevalence of wasting among under-five children by local government in 2013 and 2018 N/A: No local government area in this category.

Prevalence of wasting among under-five children by LGAs in 2013 and 2018 showed Ifedore, Ile Oluji/Oke-Igbo, and Odigbo LGAs with a prevalence of wasting ranging from 11% to 15% in 2013 dropped to zero prevalence in 2018. Also, Ose LGA with a prevalence of wasting ≥ 21% in 2013 recorded a zero prevalence in 2018. Ondo West and Akoko South West LGAs which recorded no wasting among the children in 2013 showed 16%-20% and 11%-15% wasting prevalence rates, respectively, in 2018 (Figure 2). Overall, 10 LGAs recorded zero prevalence of wasting in 2018 as compared to three LGAs in 2013, and the reduction was found to be statistically significant (p < 0.05) (Figure 2).

**Figure 3:**
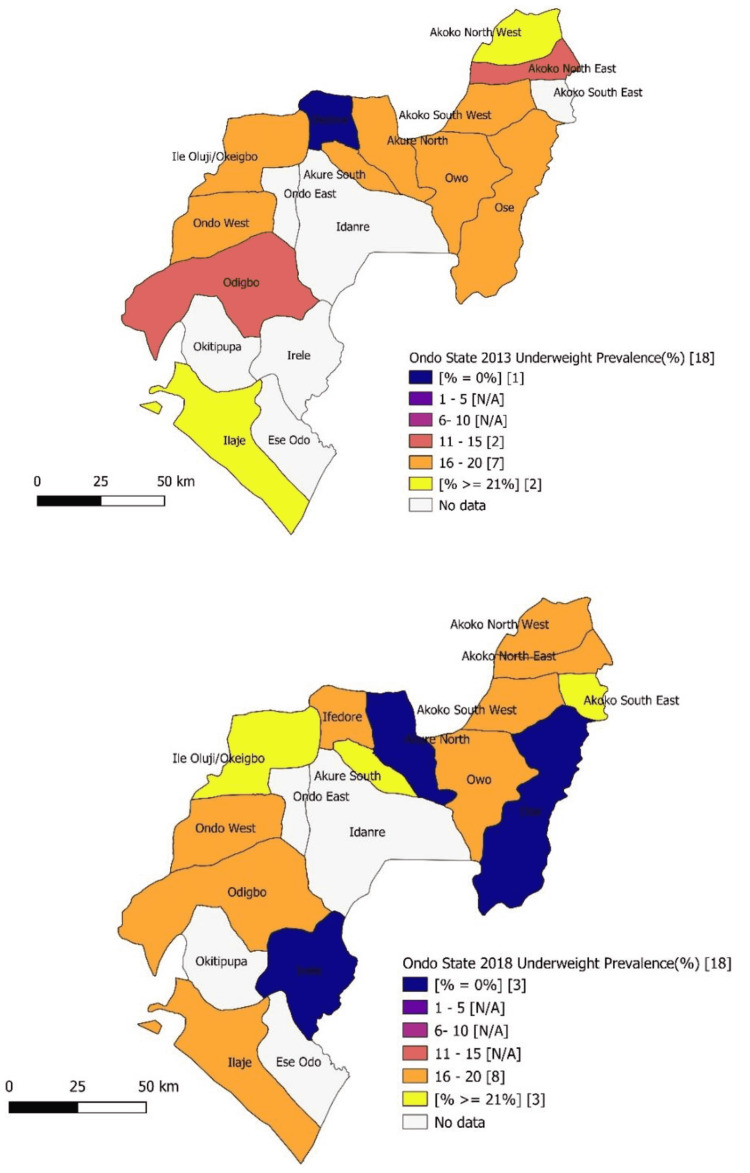
Map of the Ondo State showing a prevalence of underweight among under-five children in 2013 and 2018 N/A: No local government area in this category.

Akure North and Ose LGAs with a prevalence of underweight ranging from 16% to 20% in 2013 each recorded zero prevalence in 2018. While the prevalence of underweight also dropped from ≥ 20% in 2013 to 16%-20% in 2018 in Ilaje and Akoko North-West, Odigbo and Akoko North-East recorded an increase from 11%-15% in 2013 to 16%-20% in 2018. There was no statistically significant difference in the 2013 and 2018 prevalence of underweight in Ondo West, Owo, and Akoko South West LGAs (16%-20%) (Figure 3).

Table 2 showed the associations between children and maternal sociodemographic characteristics and indicators of malnutrition. There was a general reduction in the three indicators of malnutrition (stunting, wasting, and underweight) in 2018. Malnutrition was not associated with children's gender and maternal age in 2013 and 2018. While stunting and wasting were dependent on the age of the children and the LGAs in 2013 (p < 0.05), the prevalence of the two malnutrition indicators was not statistically significant across the children's age and LGAs in 2018. Prevalence of stunting and underweight was associated with maternal education, wealth index, residence type, place of delivery, and LGA in 2013 (p < 0.05) but was not in 2018 (p > 0.05). Two LGAs (Idanre and Ondo East) had no data recorded for 2013 and 2018, while two other LGAs (Akoko South East and Irele) had data only for 2018 but none for 2013 (Table 2).

**Table 2:** Associations between children and maternal sociodemographic characteristics and malnutrition indicators ST: Stunting; WT: Wasting; UW: Underweight; P: P-value; LGA: Local government area; NA: Data not captured in the survey.

Year	2013: Frequency (%) of malnutrition							2018: Frequency (%) of malnutrition											
	ST	PV	WT	PV	UW	PV	Total		ST	PV	WT			PV	UW	PV		Total	
Gender																			
Male	27.1	0.715	7.8	0.848	14.9	0.884	269		20.2	0.426	2.5			0.535	15.1	0.06		119	
Female	25.8		8.2		14.4		291		16		4				7			100	
Age (month)																			
0-5	13.8	0.022	19	0	12.1	0.911	58		4	0.457	8			0.293	12	0.151		25	
06-Nov	15.6		22.1		13		77		18.2		9.1				27.3			22	
Dec-23	27.1		10.4		17.7		96		23.8		2.4				4.8			42	
24-35	31.3		4.5		16.1		112		19.5		2.4				7.3			41	
36-47	32.1		0.9		14.2		106		21.3		0				12.8			47	
48-60	29.7		0.9		13.5		111		16.7		2.4				11.9			42	
Size at birth																			
Very large	27.3	0.131	6.8	0.699	4.5	0.056	44		0	0.053	0			0.499	0	0.373		20	
Larger than average	20.7		7.2		13		208		24.5		4.3				13.8			94	
Average	29.2		8.1		16.2		260		14		2.3				10.5			86	
Smaller than average	37.8		10.8		18.9		37		20		0				10			10	
Very small	27.3		18.2		36.4		11		33.3		11.1				22.2			9	
Maternal age (year)																			
15-19	15.4	0.537	15.4	0.581	15.4	0.907	13		12.5	0.543	0			0.565	0	0.855		8	
20-24	24.1		10.1		11.4		79		25		0				14.3			28	
25-29	24.8		9.4		15.4		149		19.4		3.2				12.9			62	
30-34	31.3		6		16.4		134		10.7		7.1				12.5			56	
35-39	23.3		8.6		12.1		116		20.9		2.3				11.6			43	
40-44	34		6.4		17		47		17.6		0				5.9			17	
45-49	22.7		0		18.2		22		40		0				0			5	
Maternal highest education																			
No education	39	0	15.6	0.06	26	0.001	77		25	0.495	6.3			0.35	6.3	0.672		16	
Primary	32.6		7.6		18.6		172		22.2		2.2				13.3			45	
Secondary	22.3		6.9		10.5		247		17.8		2.2				10.4			135	
Higher	10.9		4.7		6.3		64		8.7		8.7				17			23	
Wealth Index																			
Poorest		0		0.391		0			16.7	0.306	0			0.439	0	0.644		12	
Poorer	40.9		9.8		25		132		29.4		0				8.8			34	
Middle	31.1		10.4		17.8		135		20.6		2.9				14.7			68	
Richer	20.4		6.1		6.8		147		12.9		6.5				11.3			62	
Richest	15		6.2		10.3		146		14		2.3				11.6			43	
Residence																			
Urban	18.5	0	7.5	0.694	9.7	0.006	227		17.5	0.777	1			0.078	9.7	0.454		103	
Rural	31.8		8.4		18		333		19		5.2				12.9			116	
Place of delivery															7.1				
Respondent's home	38.5	0	9.2	0.925	20.2	0.026	109		28.6		0			0.026		0.663		28	
Other home	34.1		9		18.6		167		-	0.55	-							-	
Government hospital	14		6.6		8.8		136		15.2		12.1				18.2			33	
Government health center	17.5		6.3		6.3		63		17.9		2.7				11.6			112	
Government health post	25		0		0		4		-		-				-			-	
Private hospital/clinic	22.2		8.6		16		81		11.5		0				7.7			26	
Other									20		0				10			20	
LGA																			
Akoko North East	14.8	0		0.185	3.7	0	27		26.7	0.317	0			0.056	6.7	0.331		30	
Akoko North West	33.3		7.4		14.3		21		10.5		0				5.3			19	
Akoko South East	NA		0		NA		NA		23.1		0				23.1			13	
Akoko South West	7.7		NA		7.7		13		4.3		4.3				8.7			23	
Akure North	9.3		0		7		43		0		0				0			7	
Akure South	23.9		7		8.7		46		14.3		14.3				14.3			7	
Ese Odo	48.7		8.7		31		113		28.6		28.6				28.6			7	
Idanre	NA		11.5		NA		NA		NA		NA				NA			NA	
Ifedore	29.6		NA		0		27		30.8		0				7.7			13	
Ilaje	33.3		3.7		21.1		57		25		0				12.5			8	
Ile Oluji/Okeigbo	20.8		10.5		13.2		53		18.2		0				18.2			11	
Irele	NA		1.9		NA		NA		33.3		0				0			6	
Odigbo	17		NA		2.1		47		26.7		0				13.3			15	
Okitipupa	40		2.1		33.3		15		36.4		9.1				36.4			11	
Ondo East	NA		13.3		NA		NA		NA		NA				NA			NA	
Ondo West	7.1		NA		7.1		14		18.2		9.1				9.1			11	
Ose	16.4		0		9.8		61		0		0				0			16	
Total	26.4		8		14.6		560		18.3		3.2				11.4			219	

## Discussion

The Ondo State "Nutrition Rebirth" program started in 2015 adopts a comprehensive and multidimensional approach to addressing the determinants of under-five nutritional status in the State. It incorporates community mobilization and awareness creation, behavioral change communication for caregivers, production of affordable BCF from locally available food materials that proved to be quite effective in rehabilitating acutely malnourished children, vitamin supplementation and distribution of deworming tablets, live educational program to address exclusive breastfeeding, consumption of appropriate diet, and training of health workers on infant and young child feeding. In this regard, it differs from the practice in other settings that employ a noncomprehensive approach to reducing the prevalence of malnutrition [9-11].

The BCF component of the "Nutrition Rebirth" program consists of locally sourced food materials (sorghum, maize, soybean, and orange-fleshed sweet potato), which provided a cheap source of additional protein, vitamin A, and energy for the treatment of MAM among children under-five in the State. Ample anecdotal evidence from field observation and routine data showed the BCF to be effective and efficient in the management of cases of MAM in the State, and this, no doubt, contributed to the significant reduction in the prevalence of stunting and underweight in the period under review. Complementary feeding programs have been implemented in several countries and have shown positive impacts on the nutritional status of children. These include growth-monitoring practices among health workers in India nutrition education and mega-dose of vitamin A supplementation in Nepal [12-15].

There was no statistically significant difference in the mean age, size at birth, gender, maternal age, and residence of sampled children in 2013 and 2018, showing a similarity between the 2013 and 2018 sample sizes used for the study. Stunting and wasting were dependent on the age of the children in 2013 (p < 0.05) but were not statistically significant across the children's age in 2018. This is an indication of improved nutrition for the different age groups of under-five children sampled for the study during the 2013-2018 period under review. A similar finding was observed in the assessment of the prevalence of malnutrition by LGAs, and this could be attributed to the gross effect of the improved nutritional care and support to children under five years in the LGAs during the implementation of the nutrition program in the State.

However, not all the LGAs recorded an improvement in the nutritional status of the children during this period. There was no change in the nutritional status of children in few LGAs in 2018 compared to 2013, while a few others recorded an increase in the prevalence of malnutrition. (Table 2). This suggests the need for critical appraisal of the program with a view to strengthening it in those areas. Although the implementation of the comprehensive nutrition program started in Ondo State in 2015, the extent of the program utilization could differ from one LGA to the other due to some factors that are determinants of health services uptake, and that could undermine its success. Poor utilization or adoption of similar programs by mothers, e.g., growth-monitoring practices and exclusive breastfeeding, have been reported in areas of poor socioeconomic development in Africa [14]. 

Studies in Nigeria and other climes have implicated persistent negative attitudes regarding infant and young child feeding (IYCF) as a major cause of poor knowledge of health workers about similar programs both before and after the intervention, hence the poor practices and service uptake [16]. The deeply rooted sociocultural behaviors that persist among health workers shape their opinions despite previous medical or public health training and re-training [16]. The training of the State health workers on IYCF and other nutrition-related themes during the period under review would have contributed to the improvement in the knowledge and, expectedly, the attitude of the health workers, thereby facilitating improved delivery of nutrition care during the implementation of the State nutrition program [17,18]. Poor nutritional knowledge among health workers could contribute to low utilization of nutritional intervention services [19].

As expected, malnutrition was associated with maternal education, wealth index, residence type, and place of delivery, all of which are factors that affect health-care service uptake. However, this observation was only true for 2013 and not 2018. Although we cannot presently ascertain the lack of differences in nutritional status of the children relative to some of their mothers’ sociodemographic characteristics in 2018 (e.g., maternal highest education, wealth index, residence, and place of delivery), this observation could be linked to the intensive, comprehensive, and state-wide community mobilization and awareness creation about malnutrition, exclusive breastfeeding, and IYCF during the period under review. Also, there was a notable improvement in the accessibility to quality and free maternal and child health-care services provided by the government during the period, which was a motivation for mothers across the socioeconomic and demographic strata in the State to patronize government health facilities for maternal and child health care, thus reducing the effect of the differing sociodemographic characteristics that exist among them on uptake of the nutrition care services being provided through the program [19-21].

A study on the free health program in the State during this period showed that while women in the urban areas of the State were twice as more likely to deliver their babies in a health facility compared to their counterparts in rural areas (NDHS 2013), the 2016 study survey noted that the rural-urban divide in maternal residency was no longer significantly associated with higher odds of health facility delivery [22]. The persistent economic downturn in the country which affects every sector of the population could as well have contributed to the significant difference observed in the association between malnutrition and maternal demographic characteristics (education, wealth index, residence type, and place of delivery) in 2013 and not in 2018. Those who should have been able to fend for their wards may be grossly affected by economic hardship, thus posing a threat to the overall impact of the government-based complementary feeding program, which is meant to complement the parents’ efforts in nourishing their wards [23,24]. It is noteworthy that there was strong political will and improved funding for primary healthcare (PHC) activities during the period under reporting, a development that resulted in marked improvement in delivery at PHC facilities in the State (Table 1) [25,26].

The limitations of this study are ecologic fallacy and the effect of confounders. The temporal relationship cannot also be determined. This is a questionnaire-based study with data being driven by the populace. This could be a source of bias in the study as well.

## Conclusions

In conclusion, the spatial analysis of the Ondo State comprehensive nutrition program showed that the program has an impact in reducing malnutrition among children under five years. However, the program has not been completely successful in some LGAs when the nutritional status of the children pre-intervention in 2013 was compared to that of post-intervention in 2018. The Ondo State government should leverage the progress made so far and critically review the program implementation plans in LGAs with children having poor nutritional status post-intervention in order to identify the bottlenecks undermining its success.

This program highlights the effectiveness of an integrated, multi-prong, and wholistic intervention program aimed at reducing MAM burden among children under five years. This program could be utilized by other Nigerian states for tackling malnutrition and its associated challenges among children under the age of five years.
